# The association between dietary isoflavones intake and gastric cancer risk: a meta-analysis of epidemiological studies

**DOI:** 10.1186/s12889-018-5424-7

**Published:** 2018-04-17

**Authors:** Jie You, Yafei Sun, Yacong Bo, Yiwei Zhu, Dandan Duan, Han Cui, Quanjun Lu

**Affiliations:** Department of Nutrition and Food Hygiene, College of Public Health, Zhengzhou University, Zhengzhou, 450001 Henan China

**Keywords:** Gastric cancer, Dietary isoflavones intake, Meta-analysis

## Abstract

**Background:**

Isoflavones, a class of phytoestrogenic compounds, are abundant in soybeans. A number of epidemiological studies have investigated the association between dietary isoflavones intake and the risk of gastric cancer. However, the results are inconclusive. Therefore, the meta-analysis was conducted to evaluate the effect of dietary isoflavones intake on the risk of gastric cancer.

**Methods:**

Relevant studies from May 1992 to May 2017 were identified through searching PubMed and Web of Science. Additional articles were identified from the reference lists of relevant review articles. Pooled risk ratios (RRs) or odds ratios (ORs) and 95% confidence intervals (CIs) were calculated using a fixed-effects model. Funnel plot and Egger’s test were used to evaluate publication bias.

**Results:**

Seven articles reporting 12 studies were included in the current meta-analysis. We found no significant association between dietary isoflavones intake and gastric cancer risk with the highest versus the lowest categories of dietary isoflavones intake (OR = 0.97, 95% CI = 0.87–1.09, *I*^2^ = 27.5%). Subgroup analyses generally yield similar results.

**Conclusions:**

Higher dietary isoflavones intake is not associated with a decline in the risk of gastric cancer.

**Electronic supplementary material:**

The online version of this article (10.1186/s12889-018-5424-7) contains supplementary material, which is available to authorized users.

## Background

Gastric cancer, including gastric adeno-carcinoma and gastric cardia adeno-carcinoma, was the third leading cause of death in the world which accounting for 8.8% of the total cancer death, according to GLOBOCAN 2012 [1]. It is estimated that there were 951,600 new gastric cancer cases and 723,100 deaths occurred in 2012.

Epidemiological studies have suggested that high fruits and vegetables intake are inversely associated with the risk of gastric cancer [2, 3]. Isoflavones, the second metabolites during the growth of soybeans, are a group of bioactive polyphenols, which have a variety of physiological functions to human body [4]. Several epidemiological studies have investigated the relationship between dietary isoflavones intake and the risk of gastric cancer, but the results are conflicting. Therefore, we conducted the current meta-analysis to further identify the effect of dietary isoflavones intake on the risk of gastric cancer.

## Methods

### Data sources and search strategy

We searched all relevant literature from May 1992 to May 2017 using electronic databases PubMed and Web of Science, with the search terms: (“isoflavones” or “soy isoflavones” or “phytoestrogen” or “genistein” or “daidzein”) and (“gastric cancer” or “gastric tumor” or “stomach cancer” or “stomach tumor”). Additional articles were identified from the reference lists of relevant review articles.

### Inclusion criteria and exclusion criterion

Studies were selected for analysis according to the following inclusion criteria: (1) investigated the relationship of dietary isoflavones intake and gastric cancer risk; (2) the studies were either cohort or case-control studies; (3) articles reported the estimated RR/OR with their 95% CI or the indexes could be calculated. (4) articles were published in English literature. If two or more studies shared same data sets, the one with the largest sample size or the longest follow-up period was selected in the meta-analysis.

Studies were excluded if they are: (1) cell studies and animal studies; (2) reviews articles, letters to the editor and case reports; (3) isoflavones were measured with the levels in blood/urinary; (4) the RR/OR with 95% CI could not be estimated from the data in the articles.

### Data extraction

Two reviewers (Jie You and Yafei Sun) extracted the following information independently: (1) the last name of first author; (2) year publication; (3) country; (4) study design; (5) geographic area; (6) source of control; (7) participants’ characteristics (including the range of age, gender, the total number of participants, the number of cases and controls); (8) isoflavones assessment method; (9) the RRs/ORs and their 95% CIs indicating the highest versus the lowest categories of dietary isoflavones intake; (10) adjustment for covariates. Any discrepancies were definitely resolved by a third senior professor (Quanjun Lu). The quality of the selected papers was assessed by use of the Newcastle–Ottawa scale before pooled into the analysis [5]. The scores of 0–3, 4–6, and 7–9 were considered as low, moderate, and high quality, respectively. Finally, data extraction from included studies was shown in Additional file 1.

### Statistical analysis

The association between the dietary isoflavones intake and the risk of gastric cancer was calculated with the pooled ORs together with their 95% CIs. If *P* < 0.05 and/or *I*^2^ > 50%, the random-effects model was conducted. Otherwise, a fixed-effects model was used. Chi-square test and the *I*^2^ test were used to quantify the heterogeneity of the studies. Subgroup analyses by study design (the type of case-control or cohort study), gender, geographic area, source of control (based on population or hospital), sample size, dietary assessment, adjustments (family history and dietary energy intake) were also performed to find potential confounder/modifier.

Egger’s test and Funnel plots were conducted to assess the potential publication bias, and a value of *P* < 0.05 was considered as a significant difference. All statistical analyses were conducted with the software STATA version 12.0 (Stata Corporation, College Station, TX, USA).

## Results

### Characteristics of the included studies

**Fig. 1:**
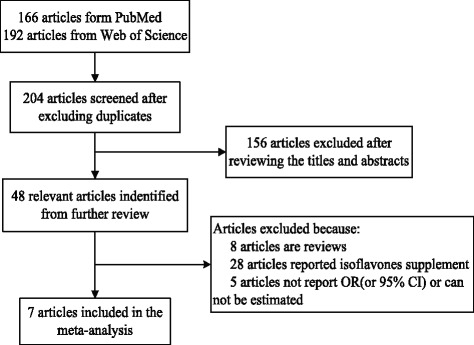
A flow diagram for selection studies and exclusion reason for the meta-analysis

The process of our articles selecting is presented in Fig. 1, a total of 204 potential relevant articles were obtained from PubMed and Web of Science. Fourty eight articles were identified by reviewing the titles and abstracts. Among these remaining 48 articles, eight articles were reviews, 28 articles reported isoflavones supplements, and two articles did not report OR/RR (or 95% CI). Finally, seven articles reporting 12 studies were included in our study, three articles with 6 cohort studies [6–8] and four articles with six case-control studies [9–12], altogether with a total of 596,553 participants among the 12 studies. Hara et al. [6] found an inverse association between isoflavone intake and gastric cancer among exogenous female hormone users. Moreover, the inverse association was also detected in the Takayama study [7]. The remaining ten studies did not find significant effect of dietary isoflavone intake on gastric cancer [6–12]. The main characteristics of the included studies are presented in Table 1.

**Table 1:** Characteristics of studies on dietary isoflavones intake and risk of gastric cancer

First author(year) County [no.]	Study design	Source of control	Age (y)	Dietary assessment	Participants (cases)	Intake comparison, high vs. Low (mg/d)	OR (95%CI) for highest vs. lowest category	NOS Score	Adjustment for covariates
Hara (2012) Japan [6]	Cohort	Population	45–74	Validated FFQ	84,881 (1249)	M: 42.3 vs. 9.2 ^a^ F: 41.8 vs. 9.4 ^a^	M: 1 (0.81–1.24) F:1.07 (0.77–1.5)	9	Age, public health center area, BMI, smoking, family history, ethanol, vegetable and fruit intake, fish intake, salt intake, and total energy intake, menopausal status
Wada (2015) Japan [7]	Cohort	Population	> 35	169-item FFQ	30,792 (678)	M: 75.5 vs. 17.6 ^a^ F: 72.6 vs. 20.1 ^a^	M: 0.81 (0.60–1.09) F: 0.60 (0.37–0.98)	9	Age, BMI, physical activity, smoking status, alcohol intake, salt intake, education, menopausal status
Rossi (2010) Italy [11]	Case-control	Hospital	22–80	Validated FFQ	777 (230)	> 34.3 vs. < 15.0	0.88 (0.53–1.46)	8	Sex, age, education, calendar year of interview, BMI, smoking intake, total energy intake
Lagiou (2004) Boston [9]	Case-control	Hospital	NR	Validated FFQ	210 (110)	2.85 vs. 0.01 ^a^	1.16 (0.73–1.84)	7.5	Age, sex, place of birth, BMI, height, education, smoking and alcohol consumption, total energy intake, fruit and vegetable intake
Petrick (2015) America [10]	Case-control	Population	30–79	104-item FFQ	1913 (589)	GCA:0.6 vs. 0.27 ^a^ OG:0.6 vs. 0.27 ^a^	GCA:1.56 (0.93–2.6) OG:1.5 (0.96–2.37)	9	Proxy status, income, education, BMI, cigarette and alcohol consumption
Woo (2014) Korea [12]	Case-control	Hospital	35–75	103-item FFQ	334 (334)	43.7 vs. 11.4 ^a^	M:0.98 (0.56–1.73) F:0.67 (0.31–1.47)	6	Age, BMI, total energy intake, H.pyloristatus, occupation, smoking and alcohol intake, meat intake, fruits and vegetables intake, physical activity
Zamora-Ros (2012) Europe [8]	Cohort	Population	35–70	Validated FFQ	477,312 (683)	> 1.1 vs. < 0.3	M: 0.77 (0.5–1.18) F: 1.05 (0.61–1.82)	9	Age, BMI, education, smoking, alcohol, physical activity, energy intake, fruit and vegetables intake, red and processed meat intake

Abbreviations: *NR* not report, *OG* gastric adenocarcinoma, *GCA* gastric cardia adenocarcinoma, *F* female, *M* male, *OR* odds ratio

^a^ Median

### Dietary isoflavones intake and risk of gastric cancer

**Fig. 2:**
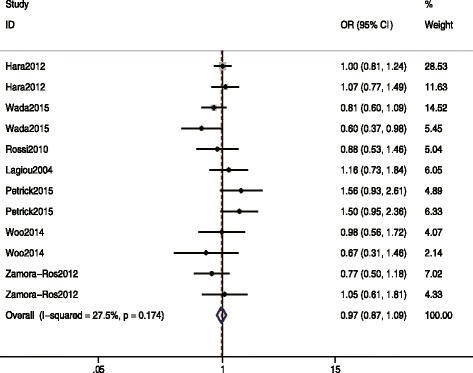
Forest plots for the association between dietary isoflavones intake and gastric cancer risk (highest vs. lowest categories)

The pooled ORs with 95%CI (highest versus lowest categories of dietary isoflavones intake) were calculated to assess the association between dietary isoflavones intake and the risk of gastric cancer. Compared to the lowest dietary isoflavones intake, the highest level was not significantly associated with the risk of gastric cancer (overall OR = 0.97, 95% CI = 0.87–1.09, *I*^2^ = 27.5%) (Fig. 2). Subgroup analysis also yield the similar results (Table 2).

**Table 2:** Subgroup analysis of dietary isoflavones intake and gastric cancer risk

Subgroup	Number of participants	Number of studies	OR (95% CI)	Analysis model	Heterogeneity test	
					*I*^2^ (%)	*P*
All studies	596,553	12	0.97 (0.87–1.09)	Fixed	27.5	0.174
Study design						
Cohort	592,985	6	0.91 (0.80–1.04)	Fixed	15.2	0.316
Case-control	3568	6	1.15 (0.93–1.43)	Fixed	15.4	0.315
Sex						
Male	196,443	4	0.91 (0.78–1.06)	Fixed	0	0.576
Female	397,210	4	0.89 (0.71–1.13)	Fixed	34.1	0.208
Mixed	2900	4	1.25 (0.98–1.59)	Fixed	8.6	0.350
Geographic area						
Europe and America	480,212	6	1.11 (0.91–1.34)	Fixed	29.2	0.316
Asia	116,341	6	0.91 (0.79–1.05)	Fixed	12.5	0.335
Source of control						
Population	594,898	8	0.98 (0.86–1.11)	Fixed	48.5	0.059
Hospital	1655	4	0.96 (0.73–1.26)	Fixed	0	0.663
Sample size						
≥ 750	595,675	7	0.99 (0.87–1.12)	Random	51.9	0.052
< 750	878	5	0.93 (0.74–1.17)	Fixed	0	0.640
Dietary assessment						
> 100 items	595,566	10	0.97 (0.86–1.09)	Fixed	37.8	0.107
≤ 100 items	987	2	1.02 (0.73–1.44)		0	0.430
Adjustments						
Family history, yes	84,881	2	1.02 (0.85–1.22)	Fixed	0	0.737
No	511,672	10	0.94 (0.82–1.09)		38.6	0.101
Dietary energy intake, yes	478,967	8	0.98 (0.85–1.12)	Random	0	0.868
No	32,705	4	0.97 (0.79–1.18)		75	0.007

### Heterogeneity analysis

The heterogeneity in the meta-analysis was relatively low (*I*^2^ = 27.5%). Meta-regression suggested that study design (case-control or cohort design), gender, geographical area, source of control (population and hospital), sample size, dietary assessment, family history and dietary energy intake for adjustments showed no significant impact on between-study heterogeneity (data not shown).

### Publication bias

**Fig. 3:**
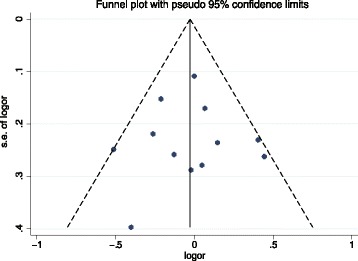
Funnel plots of dietary isoflavones intake and the risk of gastric cancer

Publication bias was evaluated with both Funnel plots and Egger’s tests. As shown in Fig. 3. The shapes of symmetrical funnel plot show little evidence of publication bias among the studies. Moreover, Egger’s test showed no significant publication bias in this meta-analysis (*t* = − 0.06, *P* = 0.957).

## Discussion

In this meta-analysis, no significant association between dietary isoflavones intake and the risk of gastric cancer was detected. The subgroup analysis was also performed to further explore the relationship, which yield the similar result.

The incidence of gastric cancer has been shown to be associated with many dietary factors [13–19]. Previous studies found that higher intake of vegetables and fruits is a protective factor against gastric cancer [3, 20]. Isoflavones, act as the role of estrogenic hormone, are rich in leguminous plants [21, 22]. Some studies showed an inverse association between dietary isoflavone intake and gastric cancer [6], however, some other studies demonstrated there is no effect of dietary isoflavone intake on gastric cancer [21, 22]. Previous studies had inconsistent findings regarding the association between isoflavones and gastric cancer risk. Therefore, it is necessary to perform a meta-analysis to further identify the associations.

Some epidemiological studies reported an inverse association between soy products and gastric risk [23, 24]. Sarah and Kweon revealed that consuming unfermented soy foods could decreased the risk of gastric cancer [25, 26]. A meta-analysis also reported that a high intake of unfermented soy foods is associated with a decreased gastric cancer risk [27]. Those findings are not agreement with our study which might be ascribed to the possible confounding effects such as salt, vegetable, fruit, and other dietary factors. After adjusting for these dietary factors, Hara [6] found no association between soy food intake and gastric cancer risk, which is consistent with our study.

In the 12 studies we selected, an inverse association between dietary isoflavones and the risk of gastric cancer was only shown in a female cohort study. This may attribute to the insufficient possible confounders adjustment. Moreover, numerous animal studies and cytology experiments have widely demonstrated the anticancer property of isoflavones [28–30] for its antioxidant and antipromotional effects [31], however, our study did not detected any protective effect of isoflavones against gastric cancer. The inconsistency might be attributed to the following reasons: first, the exposure dose and concentrations of isoflavones used in vitro and in vivo studies is precise, which is difficult to obtain through habitual dietary intake by humans [32]. Second, the composition of the gut microflora, such as *Helicobacter pylori*, may influence isoflavones absorption and metabolism and production of specific intestinal microbial catabolites which may in turn mediate their biological activities. Third, potential small existing effect may be affect by uncontrolled variability that is difficult to quantify between human beings [33].

The meta-analysis study has several advantages. First, the potential confounding factors were adjusted in all individual studies (e.g. age, BMI, smoking status and alcohol drinking). In addition, the subgroup analysis was conducted to further explore the association and strengthened the results. Moreover, the pooled results might be unbiased because no publication bias was observed.

The potential limitations of our meta-analysis should be acknowledged. First, *Helicobacter pylori* infection which is a risk factor for gastric cancer is only reported in one case-control study. Second, the dose levels of dietary isoflavones are not well defined, so the effect of dietary isoflavones intake may be masked.

## Conclusions

No significant association between dietary isoflavones intake and risk of gastric cancer was found in this meta-analysis, suggesting that dietary isoflavones intake is not associated with a decline in the risk of gastric cancer.

## Additional file

Additional file 1: Data extraction from included studies. (XLSX 15 kb)

## Abbreviations

BMI: Body mass index
CIs: Confidence intervals
F: Female
FFQ: Food frequency questionnaire
GCA: Gastric cardia adenocarcinoma
M: Male
NR: Not report
OG: Gastric adenocarcinoma
ORs: Odds ratios
RRs: Relative risks
